# The Efficacy of Non-Steroidal Anti-Inflammatory Drugs in Athletes for Injury Management, Training Response, and Athletic Performance: A Systematic Review

**DOI:** 10.3390/sports12110302

**Published:** 2024-11-06

**Authors:** Hy Pham, Frank Spaniol

**Affiliations:** Department of Kinesiology, Texas A&M University-Corpus Christi, Corpus Christi, TX 78412, USA; hpham14@islander.tamucc.edu

**Keywords:** anti-inflammatory drugs, NSAIDs, athletes, medication response, injury management, training effect, performance effect

## Abstract

(1) Background: The purpose of this systematic review is to investigate the prevalent use of non-steroidal anti-inflammatory drugs (NSAIDs) in athletes and to comprehensively review the effectiveness and the results of these medications as it relates to injury management, training response, and overall sport performance. (2) Methods: An electronic literature search was performed in accordance with the recommendations and guidelines of the Preferred Reporting Items for Systematic reviews and Meta-Analysis (PRISMA) protocol. A total of 7 randomized controlled studies met the review’s specific inclusion criteria from the 2250 studies initially identified within the PubMed database. (3) Results: In total, 346 adult female and male athletes from a variety of sporting activities and fitness levels were observed, of which 175 athletes were treated with either oral, topical, or local muscular infusion of an NSAID. Depending on study design, the outcomes focused on results obtained through physical exam findings, questionnaires, various performance metrics, and direct tissue sampling from microdialysis or biopsies. Across the 7 total studies, 2 articles focused on injured athletes and their varying pain responses with NSAIDs; 2 studies assessed the limited impact of NSAIDs on performance; and 3 articles revealed the use of NSAIDs correlating to no increases in either collagen synthesis or satellite cell activity after exercise. (4) Conclusions: The systematic review affirmed that NSAIDs can be effective for managing acute pain. However, their value appears to diminish when treating chronic injuries or if NSAIDs are expected to improve performance or have other ergogenic effects in athletes, as the aggregate data did not support such benefits. (5) Practical applications: NSAIDs can be beneficial for athletes in the right situation, but the fact that there are risks and possible disadvantageous results with their use highlights the importance of promoting appropriate expectations and the judicious use of these medications with the athletic community.

## 1. Introduction

Non-steroidal anti-inflammatory drugs (NSAIDs) are one of the most frequently used medications due to their reputation as first-line remedies for treating and managing pain as well as their generally high tolerability. The fact that they are often readily available over the counter in both oral and topical formulations makes them very convenient pharmaceutical options for people to resort to in times of need. Athletes are no exception to the common use of these medications, especially considering the prevalence of pain or discomfort that they often feel with any injury or that they can experience at any point during or after physical training and exercise or with intense play or competition. The response to treatment can depend on medication dosage and its route of administration, but overall, NSAIDs generally share the same mechanism of action through inhibition of cyclooxygenase (COX), which is the enzyme in the body that produces prostaglandins. Research has revealed that there are at least two COX isoenzymes, COX-1 and COX-2. COX-1 is present in most tissues in the body, but specific to the gastrointestinal tract, it is most known for producing prostaglandins that work to continuously protect the stomach lining by both stimulating gastric mucous production and inhibiting acid secretion. In contrast, COX-2 is generally induced through inflammatory mediators and stimuli, such as cytokines, and while this can lead to individuals subsequently experiencing unpleasant pain and swelling, the cascade of events is generally part of a larger, synchronized effort to ideally address and help resolve the conditions of illness or injury that initially triggered the inflammatory response [1].

Common over-the-counter NSAIDs such as ibuprofen, naproxen, and aspirin are non-selective COX inhibitors that indiscriminately inhibit both COX-1 and COX-2. For this reason, frequent use of these medications can cause adverse side effects such as gastrointestinal issues and bleeding. This risk exists because consistent inhibition of COX-1 can lead to unabated acid production along with insufficient mucous presence to adequately coat or protect the gastrointestinal lining from injury or ulceration. To help mitigate these gastrointestinal issues, people can still use non-selective NSAIDs, but they can instead opt for topical, non-oral formulations such as diclofenac gel or diclofenac patches that can be applied locally to the affected area(s) with less risk for systemic absorption and impact [2]. Additionally, there is the option of people getting official prescriptions for specialized NSAIDs, e.g., celecoxib or meloxicam, that are specifically designed to solely inhibit COX-2. These selective NSAIDs would thus only target the inflammatory process to help relieve pain and swelling while avoiding the undesirable side effects to the gastrointestinal system by not inhibiting COX-1.

Whether discriminately or indiscriminately inhibiting COX-2, it is logical to see why athletes would be willing to take NSAIDs since discomfort, pain, or swelling are not only unpleasant sensations for any individual, but they are also factors that could ultimately disrupt or impair an athlete’s training and sport performance. However, it is important to assess and validate these presumptions especially if there are other unintended consequences for taking these medications. As such, the overall purpose of this systematic review is to broadly investigate the prevalent use of NSAIDs in athletes and to comprehensively review the effectiveness along with the potential results and consequences that these medications can have as they relate particularly to injury management and rehabilitation, physical training and exercise, and overall athletic performance.

## 2. Materials and Methods

### 2.1. Identifying Relevant Publications

This systematic review followed the latest recommendations and guidelines per the Preferred Reporting Items for Systematic reviews and Meta-Analysis (PRISMA) protocol. A systematic literature search was performed using the PubMed database that is maintained by the U.S. National Library of Medicine at the National Institutes of Health (NIH) with the first electronic search being conducted on 29 January 2024. Keywords and terms were used both separately and in combination through the “advanced search” feature to include *non-steroidal anti-inflammatory drugs* AND *athletes* OR *athletic performance* OR *return to sport* OR *return to play*. Other keywords used include *athletes* in combination with the type and route of medication administration, such as *oral NSAIDs* OR *topical NSAIDs* OR *injectable NSAIDs*. Some common types and specific names of NSAIDs were also used within the search to include *aspirin* OR *ibuprofen* OR *naproxen* OR *diclofenac* OR *ketorolac* OR *indomethacin*. From the results of this query, initial exclusion criteria were applied for the articles that were as follows: (1) published earlier than 1 January 2000; (2) not published in English; (3) not conducted through randomized controlled trials; and (4) not readily available in full-text format.

Articles were then screened further for eligibility using information directly from their titles and abstracts with additional inclusion criteria using the Cochrane acronym PICO, which stands for *Population*, *Intervention*, *Comparison*, and *Outcome* (Table 1). The target population that was considered were functionally active athletes with no age, gender, or sport restrictions. As it relates to interventions, only studies that involved medical and non-surgical treatment of athletes with NSAIDs were included, and this systematic review required randomized controlled trials comparing a treatment group with placebo or no treatment. Lastly, regarding outcomes, all studies included in this systematic review needed to test for symptomatic or physiological changes or possible measurable changes in an athlete’s performance.

**Figure 1:**
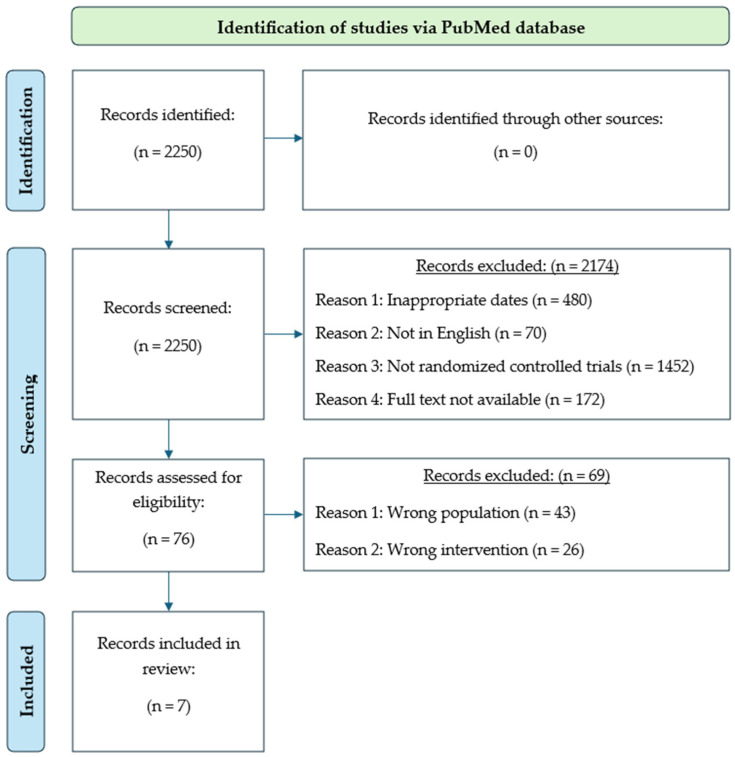
PRISMA flow chart and diagram detailing the process of article screening and assessment from initial identification to final inclusion.

Overall, the systematic literature search was performed with the goal of identifying studies that evaluated the effectiveness of the use of NSAIDs in athletes without restriction in ages, genders, fitness levels, or activities while focusing on the specific impact to injury management and rehabilitation, physical training, or athletic performance. The PRISMA flow diagram (Figure 1) was used and manually completed throughout the search process. The initial results from the “advanced search” function within the PubMed database yielded a preliminary total of 2250 articles using the aforementioned combinations of keywords and terms. A total of 1770 articles remained after excluding 480 studies that were published earlier than 1 January 2000 to ensure that only contemporary practices and findings related to NSAID usage in athletes were considered. Since there have been substantial advances in both pharmaceutical developments and the understanding of NSAID mechanisms, it was believed to be important to limit the studies to this specific timeframe to reflect modern clinical standards and recommendations. Subsequently, an additional 70 articles were removed because they were not published in English, and 1452 articles were additionally excluded as they were not randomized controlled studies. At this point, 248 studies remained, but it was then discovered that 172 of these articles were not available in full text for review. As such and after removing them, 69 articles remained from the original total of 2250, and from this last pool of studies, 62 did not meet final inclusion criteria due to a combination of having inappropriate testing populations or unfitting interventions based on the needs of this systematic review. This ultimately resulted in 7 articles left for full-text assessment and final inclusion.

### 2.2. Assessing Study Quality

The final 7 studies that met all inclusion criteria to be included in this systematic review were formally assessed using the Oxford quality scoring system, also known as the Jadad scale, which was created to assist in evaluating overall study quality. In review, studies that are being assessed with this methodology can have a total score ranging from 0 to 5 points, where studies scoring between 0 and 2 points are considered of low quality, while those scoring between 3 and 5 points are consistent and indicative of high-quality studies. There are three distinct categories of scoring based on randomization, blinding, and dropout. An article receives 1 point if it mentions randomization and another point if it includes and describes the appropriate randomization method. Similarly, an article can receive 1 point if double blinding is discussed, along with an additional point when the blinding methodology is included. The maximum score for each randomization and blinding category is 2 points, but if the methods of randomization or blinding described within the study are deemed to be inappropriate, 1 point can be deducted from the respective randomization or blinding scores. For the third and last category of dropout, an article receives 1 point if there is any discussion of participant withdrawal or dropout [3].

In applying this scoring system to the 7 articles included in this systematic review, 2 studies were found to be of low quality while 5 were considered high-quality studies. A table displaying the results of the Oxford quality scoring system as it applies to the 7 studies assessed in this systematic review is included below (Table 2).

## 3. Results

All seven articles within this systematic review were thoroughly reviewed and had pertinent study characteristics and information compiled into a data table to include the year that the study was published, the characteristics and number of participants of each study, general details of the study design, relevant measures used, and the outcomes (Table 3). In total, across all seven studies, 346 adult male and female athletes ages 18 years and older from a variety of sporting activities and fitness levels were observed, of which 175 athletes were treated with either oral, topical, or local muscular infusion of an NSAID. The specific NSAIDs used with the participants across the seven studies include treatment doses of a topical 1% diclofenac patch, 10% diclofenac gel, 100 milligrams (mg) of oral indomethacin, 45 mg of indomethacin via infusion, and 200 mg to 400 mg of oral ibuprofen.

Depending on study design, the period of post-treatment analysis ranged from 1 day to 12 weeks and involved comparisons from the results obtained through physical exam, symptom- and function-oriented questionnaires, various performance metrics, or direct soft tissue sampling from microdialysis or muscle biopsies.

Of the seven studies in this systematic review, two specifically involved assessing athletes with known injuries and their treatment response to NSAIDs. In one of these two studies, the focus was primarily on both male and female recreational athletes with chronic Achilles tendinopathy and their treatment response to topical diclofenac gel. In this study, after 4 weeks of treatment, no significant differences were ultimately observed between the placebo and diclofenac groups in any of its outcome measures, including numeric pain rating, patient-reported symptom changes, pressure–pain threshold, tendon stiffness, or Victorian Institute of Sports Assessment—Achilles (VISA-A) questionnaire scores at 4 and 12 weeks [4]. In the other study that assessed injured athletes, the focus was strictly on non-chronic, minor injuries and the athletes’ pain response throughout their treatment and management with an NSAID. This study involved both male and female athletes who had recently sustained an acute sports-related, soft-tissue injury within 3 days prior to the start of the randomized controlled trial. They were treated with either topical diclofenac patches or a placebo daily for 2 weeks. The athletes’ pain intensity was recorded every day and during clinic visits using pain relief scales, and overall, the results showed that those treated with diclofenac patches had significantly reduced pain compared to the placebo group [2].

Of the other five studies within this systematic review, three articles specifically assessed how athletes physiologically responded to NSAIDs by performing direct tissue analysis before and after completion of their assigned exertional activities. One study involved healthy male runners who were training for a marathon. They were given either oral indomethacin or a placebo starting from 72 h before the start of their 36 km run through 72 h after their completion of it. Based on microdialysis samples taken directly from the patellar tendon, the placebo group showed unchanged prostaglandin E2 (PGE2) levels with significantly increased levels of procollagen type I N-terminal propeptide (PINP) concentrations, which serves as a surrogate marker indicative of collagen synthesis. Conversely, the group treated with indomethacin showed significantly decreased PGE2 levels with no increase in collagen synthesis [5]. In another study that assessed the physiological impact of NSAIDs after activity, male endurance-trained athletes were also given either oral indomethacin or a placebo and were told to complete a 36 km run. However, the treatment was given from 4 days prior through 8 days after completion of the run, and instead of tissue samples obtained through microdialysis, muscle biopsies were conducted before and on days 1, 3, and 8 after the run, specifically assessing the amount of satellite cell activity. Ultimately, the results revealed that the placebo group had increased satellite cell activity post-exercise, whereas the group treated with NSAIDs had no such increase [8]. Similarly, another study within this group of articles also assessed satellite cell activity, but it did so from a very different study design. Instead of runners, it took male recreational athletes who were considered to be well-trained, as defined by conducting at least 6 h of physical training per week. However, an additional inclusion criterion for its participants was having the requirement of not doing any leg resistance training within the last year. The athletes were ultimately tasked with completing 200 maximal eccentric contractions of each leg, with one leg treated with an infusion of indomethacin while the other leg served as the control. In comparing muscle biopsy samples before and 8 days after the exercise activity, there was a significant increase in satellite cell proliferation in the control leg, while no increase in satellite cell activity was observed in the NSAID-treated extremity [9].

The final two studies of this systematic review assessed the potential impact of NSAIDs specifically from the perspective of athletic performance. In one study, well-conditioned endurance runners completed a muscle-damage protocol that consisted of both concentric and eccentric exercises. They were then given either ibuprofen or a placebo, and two days later, they completed a treadmill running test. Both groups showed reduced endurance performance 48 h after completing the muscle-damage protocol, but no significant difference was ultimately observed between the test groups [5]. The last study in this systematic review also focused on healthy male long-distance runners, and they were given either oral ibuprofen or a placebo 15 min before and 5 h into a 42 km trail run. In the end, it was revealed that the NSAID users had fewer oxidative stress markers but otherwise no significant difference in race performance nor with muscle damage indicators such as creatine kinase [6]. All results in this systematic study have been organized in Table 3.

## 4. Discussion

### 4.1. Interpreting the Findings

The purpose of this systematic review was to review the effectiveness and impact of NSAIDs as they relate to injury management, training response, and overall sport performance in athletes. Based on the results across the seven studies in this systematic review, there are some key interpretations that can be made when comparing the data between the treatment and control groups within these seven randomized controlled studies. In the same corresponding order in which the articles were discussed in the previous section, topical diclofenac gel was shown to ultimately not provide any superior clinical outcome compared to placebo in treating and managing chronic Achilles tendinopathy over a 4-week period [4]. On the other hand, diclofenac patches were found to be significantly effective at treating non-chronic, acute pain over the course of just two weeks for athletes who had experienced recent, minor sports injuries [2]. Between these two articles, it would suggest that NSAIDs have limited value for treating pain from chronic conditions, but they nevertheless remain a safe and effective option for successful pain management of minor, acute sports injuries.

As it pertains to the impact of NSAIDs on the body’s physiological response after exercise, one study showed that the intake of indomethacin significantly blocked the production of prostaglandins and also significantly suppressed the natural adaptive increase in collagen synthesis that usually occurs as a response to exercise [5]. In two other studies, it was further demonstrated that indomethacin significantly inhibited the normal increase in satellite cell activity after exercise as well [8,9]. Especially when considering that satellite cells are essential for proper recovery and healing from exercise, these three studies not only suggest that the cyclooxygenase (COX) pathway is associated with the regulation of collagen synthesis and satellite cell proliferation but also that NSAIDs could potentially, therefore, also impair muscle recovery and post-exercise adaptive processes [1]. Whether this would subsequently lead to risks of decreased performance in the future though was admittedly not the scope of these studies. However, regarding NSAIDs and whether they would more immediately affect an athlete’s performance, there were two articles in this systematic review that studied long-distance runners and how they fared on a treadmill running test or on a 42 km run after being given either ibuprofen or a placebo. Both studies ultimately demonstrated that NSAIDs did not enhance physical performance in either circumstance [5,6].

### 4.2. Systematic Review Limitations

Systematic reviews are most helpful in summarizing the evidence and outcomes of various intervention trials, but as with any review, there are some key limitations. In this systematic review, particularly, the stated purpose of the review was admittedly broad. This was done intentionally to be able to assess the wide range of impact that NSAIDs can have on athletes, as evidenced by their common and prevalent use, whether appropriate or not. However, a broad purpose or a broadly stated review question will generally lead to the risk of having wide inclusion criteria as well, and this systematic review was no exception to that tendency and vulnerability. As a result of having a broad purpose, there was ultimately inclusion of studies that varied widely in the characteristics of the population being studied, the interventions performed, the types of measures used for comparison, and the outcomes. Narrowing the scope of the review’s purpose and its inclusion criteria would have led to more direct and possibly more reliable comparisons across all included studies.

It is also fully acknowledged that utilizing multiple databases in systematic reviews can help ensure comprehensive coverage. However, in this systematic review, PubMed was the only database that was used. The decision to prioritize PubMed for this review was primarily due to its specific focus on biomedical literature, which aligns closely with the topic of NSAIDs and their effects on athletes. Given the scope and inclusion of clinical trials and medical studies published in articles from multiple journals, PubMed was considered the most relevant database to facilitate access to a broad range of high-quality studies without significant duplication risk. Even so, a backward search of bibliographies could have potentially uncovered additional relevant studies, but the decision was made to only focus on studies that met the strict criteria outlined in the PRISMA guidelines, thus ensuring that the included studies were identified through a standardized search methodology rather than more opportunistic methods that could introduce bias.

Another limitation of this systematic review is that the identification and screening process of articles is usually conducted by at least two reviewers, but this review only had one. As such, no inter-rater reliability could be established, not only for the selection of studies but also for data extraction and data entry. It is fully recognized that involving only one reviewer can introduce a risk of bias and affect the consistency of outcomes, and while this systematic review was thoroughly supervised, the decision to have only one reviewer was ultimately driven by resource limitations. To mitigate this limitation to the fullest, there was implementation of strict adherence to the PRISMA guidelines along with employment of objective inclusion and exclusion criteria to help minimize subject judgment as much as possible.

Overall, by including additional reviewers and having a query with greater inclusion of studies across additional research databases or by narrowing the scope of this systematic review in future iterations, greater reliability along with stronger and more validated insights can certainly be facilitated. However, even so, there are still appropriate conclusions and practical applications that can be extrapolated from this review in its current state.

## 5. Conclusions

Despite its limitations, this systematic review did investigate the wide uses of NSAIDs. From selecting a total of seven articles in accordance with the latest recommendations and guidelines per the PRISMA protocol, the systematic review was able to review the effectiveness along with the potential results and consequences that NSAIDs can have on athletes, particularly as it relates to injury management and rehabilitation, physical training and exercise, and athletic performance. Overall, this systematic review affirmed that NSAIDs can be effective for managing acute pain in recent sports injuries. However, the beneficial value of NSAIDs does seemingly diminish when it comes to treating more chronic injuries, as evidence shows that symptomatic relief and functional improvement with NSAIDs for such conditions are negligible. Lastly, there has been a belief that NSAIDs could possibly be ergogenic and provide athletic performance enhancement because of their anti-inflammatory and analgesic properties. However, it is important to make the critical distinction between “performance enhancing” versus “performance enabling”. While more research beyond this systematic review is admittedly needed to support whether the latter is applicable to NSAIDs, this review provided data that NSAIDs are not performance-enhancing agents. In fact, not only does the data in the included studies show that NSAIDs do not improve athletic performance, but there is also evidence to suggest that the use of NSAIDs before, during, or after major physical activity can potentially impair an athlete’s physiological response to exercise as it specifically relates to recovery and training adaptations.

## 6. Practical Applications

As it has been stated, the use of NSAIDs can be very prevalent in athletes since these medications are often readily available, can provide positive pain relief, and are generally well tolerated, with low risk of major adverse side effects. However, at the same time, resorting to NSAIDs is not a completely benign intervention with zero risk. While NSAIDs can certainly be beneficial for athletes in the right situation, the fact that there are real risks and possible disadvantageous results with their use highlights the importance of promoting the appropriate use of these medications throughout the athletic community. As such, proper education and awareness amongst athletes, parents, coaches, and healthcare professionals is vital for helping ensure that an appropriate risk and benefit analysis is performed every time the use of NSAIDs is being considered. Doing so will help mitigate the prevalence of inappropriate expectations, medication misuse, and ultimately the risks of potential harm and other unintended consequences.

## Figures and Tables

**Table 1:** Inclusion criteria used for this systematic review according to PICO.

INCLUSION CRITERIA FOR SYSTEMATIC REVIEW	
**PICO**	**Targeted Characteristics & Elements**
Population	Functional athletes with no age, gender, or sport restrictions
Intervention	Medical/non-surgical management with NSAIDs only
Comparison	Control group with placebo or no treatment
Outcome	Symptomatic or physiological changes or changes in performance metrics

**Table 2:** Oxford Quality Scoring System, also known as the Jadad Scale, assessing study quality.

OXFORD QUALITY SCORING SYSTEM					
Article Source/Year	Randomization Score	Blinding Score	Dropout Score	Total Score	Quality of Study
Bussin et al., 2021 [4]	2	2	1	5	HIGH
Christensen et al., 2011 [5]	2	0	1	3	HIGH
Da Silva et al., 2015 [6]	2	2	1	5	HIGH
de Souza et al., 2024 [7]	2	1	1	4	HIGH
Galer et al., 2000 [2]	1	1	1	3	HIGH
Mackey et al., 2007 [8]	1	0	1	2	LOW
Mikkelsen et al., 2009 [9]	1	0	0	1	LOW

**Table 3:** Summary of compiled characteristics and results from the seven included studies.

SUMMARY DETAIL OF ARTICLES CHOSEN FOR SYSTEMATIC REVIEW					
Source/Year	Participants	N	Design	Relevant Measures	Outcomes
* Bussin et al., 2021 [4]	Male and female recreational athletes with chronic Achilles tendinopathy	67	Randomized controlled trial (RCT) with topical diclofenac or placebo for 4 weeks	Numeric pain rating, patient-reported symptom changes, pressure pain threshold, tendon stiffness, and VISA-A scores at 4 and 12 weeks	No statistically significant differences were observed between treatment and placebo groups.
* Galer et al., 2000 [2]	Male and female athletes with an acute sports-related, soft tissue injury within 3 days prior to study	213	Multicenter RCT with topical diclofenac or placebo applied daily for 2 weeks	Pain intensity recorded daily and during clinic visits using pain relief scales.	Those treated with diclofenac patches had statistically significantly reduced pain levels compared to placebo.
** Christensen et al., 2011 [5]	Healthy male, experienced runners training for a marathon	15	RCT with runners tasked to complete 36 km run and being given oral indomethacin or placebo starting from 72 h pre-activity through 72 h post-exercise	Collagen synthesis using PINP levels and prostaglandin E2 (PGE2) concentrations were assessed in patella tendon before and 72 h after exercise.	Placebo group showed unchanged PGE2 levels with statistically significant increase in collagen synthesis. NSAID group showed significantly decreased PGE2 levels with no increase in collagen synthesis.
** Mackey et al., 2007 [8]	Healthy male, endurance-trained athletes	11	RCT with runners tasked to complete 36-km run and being given oral indomethacin or placebo from 4 days prior through 8 days after the run	Satellite cell activity measured from muscle biopsies collected before and on days 1, 3, and 8 after the run	Placebo group had statistically significantly increased levels of satellite cell activity post-exercise. The NSAID group showed no increase.
** Mikkelsen et al., 2009 [9]	Healthy male recreational athletes, well-trained (6-h training/week) but no leg resistance training within past year	8	RCT with participants to complete 200 maximal eccentric contractions of each leg, with one leg treated with infusion of indomethacin while the other leg served as the control.	Satellite cell activity measured from muscle biopsies before and 8 days after the exercise	There was a statistically significant increase in satellite cell proliferation in the control leg, while no increase in satellite cell activity was observed in the NSAID-treated leg.
*** Da Silva et al., 2015 [5]	Healthy male, well-conditioned endurance runners	20	RCT with oral ibuprofen or placebo given after muscle-damage protocol of concentric and eccentric exercises but prophylactically before treadmill run test	Time until self-reported fatigue via treadmill running test	Both groups experienced reduced endurance performance 48 h after muscle-damage protocol, but no statistically significant difference observed between test groups.
*** de Souza et al., 2024 [6]	Healthy male, long-distance runners	12	RCT with oral ibuprofen and placebo given 15 min before and 5 h into 42-km trail run	Oxidative stress markers, muscle damage indicators, run time	Ibuprofen users showed less oxidative stress markers but otherwise no statistically significant difference in creatine kinase levels or race performance.

* Identified articles assessing the impact of NSAIDs to pain in injured athletes. ** Identified articles assessing the impact of NSAIDs to post-exercise physiological response. *** Identified articles assessing the impact of NSAIDs on athletic performance.

## Data Availability

No new data was created or analyzed in this study. Data sharing is not applicable to this study.
